# Outcome Analysis of Transition From Peritoneal Dialysis to Hemodialysis: A Population-Based Study

**DOI:** 10.3389/fmed.2022.876229

**Published:** 2022-06-02

**Authors:** Ming-Hsien Tsai, Yun-Yi Chen, Tsrang-Neng Jang, Jing-Tong Wang, Yu-Wei Fang

**Affiliations:** ^1^Division of Nephrology, Department of Internal Medicine, Shin-Kong Wu Ho-Su Memorial Hospital, Taipei, Taiwan; ^2^Department of Medicine, Fu Jen Catholic University School of Medicine, Taipei, Taiwan; ^3^Department of Research, Shin-Kong Wu Ho-Su Memorial Hospital, Taipei, Taiwan; ^4^Institute of Hospital and Health Care Administration, National Yang Ming Chiao Tung University, Taipei, Taiwan; ^5^Department of Internal Medicine, Shin-Kong Wu Ho-Su Memorial Hospital, Taipei, Taiwan

**Keywords:** hemodialysis, peritoneal dialysis, peritoneal dialysis technique failure, mortality, hospitalization, major adverse cardiac outcomes

## Abstract

If a technical failure occurs during peritoneal dialysis (PD), the patients undergoing PD may be transitioned to hemodialysis (HD). However, the clinical outcomes of patients who have undergone such a transition are under studied. This study assessed whether patients undergoing HD who have transitioned from PD have the same clinical outcomes as HD-only patients. This research was a retrospective cohort study by searching a National Health Insurance research database for data on patients in Taiwan who had undergone HD between January 2006 and December 2013. The patients were divided into two groups, namely a case group in which the patients were transitioned from PD to HD and a HD-only control group, through propensity score matching at a ratio of 1:4 (*n* = 1,100 vs. 4,400, respectively). We used the Cox regression model to estimate the hazard ratios (HRs) for all-cause death, all-cause hospitalization, infection-related admission, and major adverse cardiac events (MACE). Those selected patients will be followed until death or the end of the study period (December, 2017), whichever occurs first. Over a mean follow-up of 3.2 years, 1,695 patients (30.8%) died, 3,825 (69.5%) required hospitalization, and 1,142 (20.8%) experienced MACE. Patients transitioning from PD had a higher risk of all-cause death (HR: 1.36; 95% CI: 1.21–1.53) than HD-only patients. However, no significant difference was noted in terms of MACE (HR: 0.91; 95% CI: 0.73–1.12), all-cause hospitalization (HR: 1.07; 95% CI: 0.96–1.18), or infection-related admission (HR: 0.97, 95% CI: 0.80–1.18) between groups. Because of the violation of the proportional hazard assumption, the piecewise-HRs showed that the risk of mortality in the case group was significant within 5 months of the transition (HR: 2.61; 95% CI: 2.04–3.35) not in other partitions of the time axis. In conclusion, patients undergoing HD who transitioned from PD had a higher risk of death than the HD-only patients, especially in the first 5 months after transition (a 161% higher risk). Therefore, more caution and monitoring may be required for patients undergoing HD who transitioned from PD.

## Introduction

Taiwan has a high burden of chronic kidney disease (CKD) (1). In a comparison of international data from the United States Renal Data System in 2016, Taiwan was reported to have the highest incidence [0.455/1,000 person-years (PY)] and prevalence (0.32%) of end-stage renal disease (ESRD) requiring renal replacement therapy among surveyed countries, and it maintained high levels of incidence and prevalence in subsequent years (2, 3). The trend of choosing peritoneal dialysis (PD) as treatment for ESRD has gradually decreased in Taiwan, accounting for only 6–8% of dialysis treatments from 2000 to 2018. This may be due to a decrease in new-onset PD from 14.2% in 2007 to 9.4% in 2018. Because of this decrease, hemodialysis (HD) has gained prevalence (4).

Compared with HD, PD offers several clinical benefits. First, patients undergoing PD have been reported to have a better 3-year survival rate, although the age-adjusted life span for both treatments was similar (2). Second, the degree of residual kidney function preservation and quality of life has been reported to be more favorable in PD than in HD (5, 6). Third, PD is more cost-effective than HD (7, 8). Accordingly, PD has been widely recognized and employed as the first therapy of choice (9).

However, several limitations and risks have been reported for PD that lead to patients requiring a transition to HD. The most prevalent risk is PD-related peritonitis, which is the reason for 28–35% of PD dropouts (10). In addition, gradual loss of residual kidney function and progressive reduction of peritoneal function resulting from PD may cause insufficient clearance and ultrafiltration (11). These limitations often negate the initial benefits of PD, and approximately 35% of patients undergoing PD drop out of the therapy and require transition to HD (12).

Because transition from PD to HD is inevitable for patients with ESRD, no study has yet analyzed outcomes by using a real-world dataset. In this study, we compared the outcomes of patients who transitioned from PD to HD with those of patients undergoing HD-only using a nationwide population-based dataset.

## Materials and Methods

### Data Sources and Research Samples

We used a CKD sub-database from the National Health Insurance Research Database (NHIRD) maintained by the Health and Welfare Data Science Center of the Ministry of Health and Welfare of Taiwan. The diagnosis of CKD includes 124 ICD-9-CM codes that were verified officially in the Chronic Kidney Disease Prevention Technology Research Project conducted by the Health Promotion Administration, Ministry of Health and Welfare (13). Since its launch in 1995, the NHIRD has compiled records of 99% of Taiwan’s 23 million citizens who have enrolled in the Taiwan National Health Insurance program (14). Before they are released from the NHIRD, data are deidentified and identifying information is encrypted. The deidentified, personal information retained in the data are date of birth, sex, area of residence, diagnostic codes, medical procedures, and drug prescriptions. Before 2015, diseases listed in the NHIRD were defined according to International Classification of Diseases, Ninth Revision, Clinical Modification (ICD-9-CM) codes. After 2015, diseases were defined according to International Classification of Diseases, Tenth Revision, Clinical Modification (ICD-10-CM) codes. This study was exempted from full ethical review and was approved by the institutional review board of Shin-Kong Wu Ho-Su Memorial Hospital (IRB approval number: No. 20200806R). Informed consent was waived by the Ethics Committee of Shin-Kong Wu Ho-Su Memorial Hospital because the personal information of all beneficiaries listed in the NHIRD was deidentified.

### Study Design and Population

**FIGURE 1:**
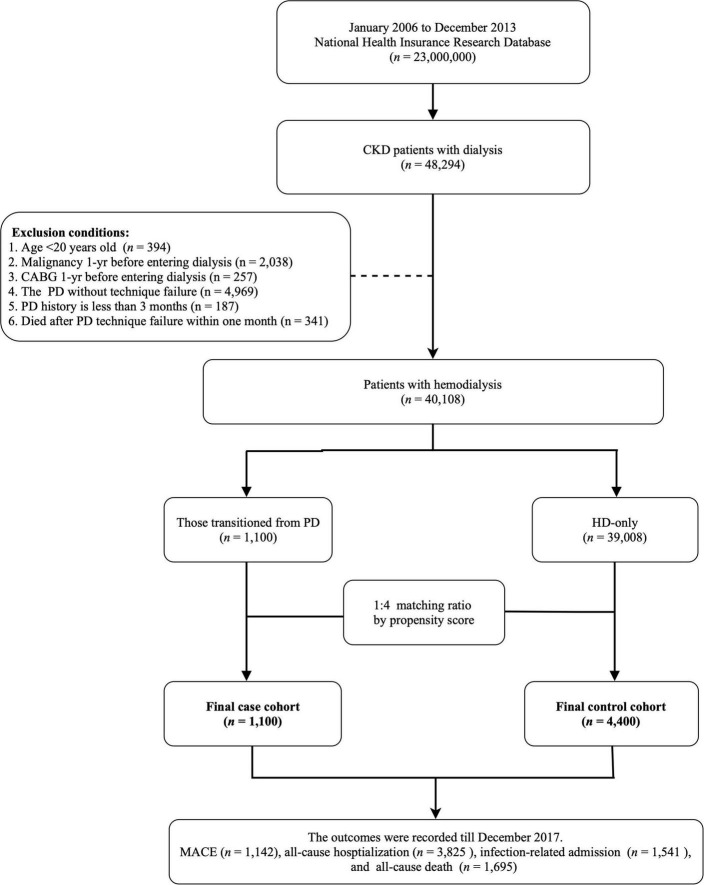
Schematic of patient enrollment. CKD, chronic kidney disease; PD, peritoneal dialysis; CABG coronary artery bypass graft; HD, hemodialysis; MACE, major adverse cardiovascular events.

This study was designed as a population-based longitudinal cohort study. The target population was patients who newly entering chronic dialysis (receiving dialysis more than 3 months) from January 1, 2006, to December 31, 2013 (*n* = 48,294). Patients who were under 20 years of age, were undergoing PD, and were given a diagnosis of malignancy or who underwent coronary artery bypass graft (CABG) 1 year prior to beginning dialysis were excluded. Moreover, patients who transitioned from PD to HD with a PD history of less than 3 months or who died within a month of dialysis transition were also excluded. A total of 40,108 patients undergoing dialysis were included in the analysis. To reduce baseline differences between study groups, we used 1:4 propensity score matching for variables age, sex, and Charlson comorbidity index score (15). Accordingly, our analysis included patients undergoing HD who had transitioned from PD (*n* = 1,100) and patients in a matched comparison group (*n* = 4,400; Figure 1). The index day for the first group was 1 month after the transition from PD to HD. The index day for the HD-only group was the day when they started to undergo chronic dialysis, plus the dialysis vintage of the matching case. Those selected patients will be followed until death or the end of the study period (December 31, 2017), whichever occurs first.

Baseline comorbidities, including hypertension, diabetes mellitus, coronary artery disease (CAD), chronic heart failure (CHF), atrial fibrillation, peripheral vascular disease (PVD), stroke, chronic obstructive pulmonary disease (COPD), hyperlipidemia, and polycystic kidney disease, were defined as present if the patient had at least three outpatient diagnoses or one inpatient diagnosis within the year preceding the index date (Supplementary Table 1). Use of drugs, including renin–angiotensin system blockers (RASB), beta-blockers, calcium channel blockers (CCB), anticoagulants, dipeptidyl peptidase-4, and lipid-lowering agents, was defined as present if the patient had used the drug for least 3 months within 1 year preceding the index date (Supplementary Table 2).

### Definition of Outcomes

The primary outcomes of this study were all-cause mortality, all-cause hospitalization, infection-related admission, and major adverse cardiac events (MACE), which comprises myocardial infarction, cerebrovascular disease, heart failure, and arrhythmia (Supplementary Table 1). Data were analyzed from the index date to the first instance of the desired outcome (as previously defined) or to the end of the study period (December 31, 2017).

### Statistical Analysis

Continuous data of the baseline characteristics are expressed as mean ± standard deviation, and categorical data are expressed as counts with proportions. The differences between the groups were determined using Chi-squared tests and *t* tests for proportions and continuous variables, respectively. The Kaplan–Meier method was employed for estimating and plotting event-free curves, which were then tested using a log-rank test. A Cox proportional-hazard model for clustered data was used to estimate the hazard ratios (HRs), and a 95% confidence interval (CI) was calculated for the risk of clinical outcomes. Both crude-adjusted and multivariable-adjusted analyses were performed. The assumption of proportional hazard was tested for the interaction between time and the variables. If the assumption was violated, the time axis was partitioned for the further analysis. Moreover, death was considered a competing risk for the development of desired outcomes in our analysis.

All statistical analyses were performed using SAS software, version 9.4 (SAS Institute, Cary, NC, United States). For all tests, two-sided *p* values < 0.05 indicated statistical significance.

## Results

### Patient Characteristics

In the present study, we enrolled 5,500 patients undergoing HD (Figure 1). Among these patients, 1,100 had transitioned from PD to HD and 4,400 were HD-only. The two groups were matched using propensity scores. The differences in the baseline characteristics between groups are presented in Table 1. Several significant differences in comorbidities, such as for hypertension, diabetes mellitus, atrial fibrillation, stroke, COPD, and hyperlipidemia (all *p* < 0.05), were identified between the groups. Moreover, the patients who had previously undergone PD had higher instances of the use of RASB, beta-blockers, CCB, anticoagulants, dipeptidyl peptidase-4, and lipid-lowering agents (all *p* < 0.05).

**TABLE 1:** Baseline characteristics of study population on hemodialysis after matching.

	Transitioned from PD	HD-only	*P* value
	**(*n* = 1,100)**	**(*n* = 4,400)**	
**Age (years)**			0.949
<65	836 (76.0)	3,340 (75.9)	
≤65	264 (24.0)	1,060 (24.1)	
**Gender**			0.859
Male	640 (58.2)	2,547 (57.9)	
Female	460 (41.9)	1,853 (42.1)	
Dialysis vintage	2.83 ± 0.57	2.83 ± 0.57	1
CCI score	1.35 ± 1.10	1.33 ± 1.08	0.57
**Baseline comorbidities**			
Hypertension	568 (51.6)	1,853 (42.1)	< 0.001
Diabetes mellitus	422 (38.4)	1,507 (34.2)	0.01
CAD	191 (17.4)	725 (16.9)	0.48
CHF	115 (10.5)	388 (8.8)	0.092
Atrial fibrillation	28 (2.6)	54 (1.2)	0.001
PVD	65 (5.9)	253 (5.8)	0.839
Stroke	109 (9.9)	331 (7.1)	0.001
COPD	81 (7.4)	236 (5.4)	0.01
Hyperlipidemia	180 (16.4)	546 (12.4)	< 0.001
Polycystic kidney	1 (< 0.1)	16 (< 0.1)	0.223
**Prescriptions**			
RASB	448 (40.7)	1,251 (28.4)	< 0.001
Beta-blocker	449 (40.8)	1,314 (29.9)	< 0.001
CCB	520 (42.3)	1,636 (37.2)	< 0.001
Anti-cogulants	369 (33.6)	951 (21.6)	< 0.001
DPP4	121 (11)	330 (7.5)	< 0.001
Lipid-lowering agents	294 (26.7)	809 (18.4)	< 0.001

*HD, hemodialysis; PD, peritoneal dialysis; CCI, charlson comorbidity index; CAD, coronary artery disease; CHF, congestive heart failure; PVD, peripheral vascular disease; COPD, chronic obstructive pulmonary disease; RASB, renin-angiotensin system blockades; CCB, calcium channel blockers; DPP4, dipeptidyl peptidase-4.*

### All-Cause Deaths Between Groups

**FIGURE 2:**
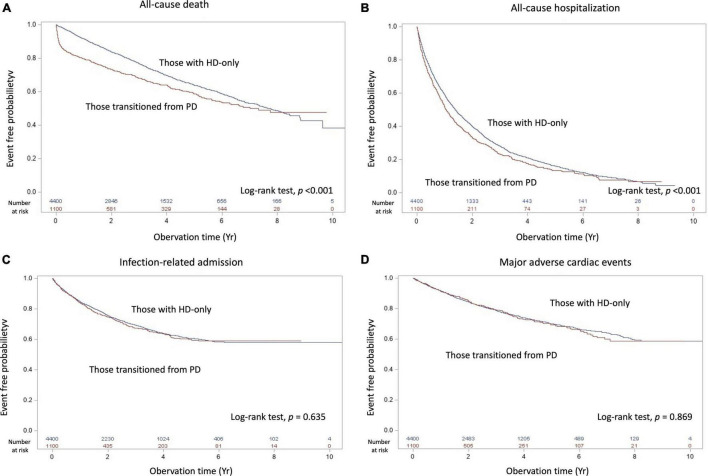
Kaplan–Meier cumulative event-free plots of **(A)** all-cause death, **(B)** all-cause hospitalization, **(C)** infection-related hospitalization, and **(D)** major adverse cardiac events in the study population based on having experienced peritoneal dialysis; the results of log-rank tests were χ^2^ = 33.01 (*p* < 0.001), χ^2^ = 12.89 (*p* < 0.001), χ^2^ = 0.22 (*p* = 0.635) and χ^2^ = 0.03 (*p* = 0.869), respectively. Abbreviation: HD, hemodialysis; PD, peritoneal dialysis.

Within the mean follow-up time of 3.2 years, all-cause death differed between the transition from PD to HD and HD-only groups, with 385 patients [incidence, 0.126/person-year (PY)] and 1310 patients (incidence, 0.089/PY) dying, respectively (Table 2). Table 3 shows no significant difference of the causes of death between groups (*p* = 0.748). The deaths resulted from cancer, sepsis, cardiovascular disease and dialysis between the transition from PD to HD and HD-only groups were 22 (5.7%) versus 76 (5.8%); 43 (11.2%) versus 152 (11.6%); 97 (25.2%) versus 315 (24 %); and 85 (22.1%) versus 326 (24.9%), respectively. The Kaplan–Meier event-free curves for all-cause deaths are presented in Figure 2A. The log-rank test indicated that the difference between the two groups was significant (χ^2^ = 33.02, *p* < 0.001). However, the two survival curves came close as time went on, indicating it may violate the proportional hazard assumption.

**TABLE 2:** Outcomes between hemodialysis patients with/without previous peritoneal dialysis.

Outcomes	Transitioned from PD		HD-only		Transitioned from PD vs. HD-only			
	Events	IR	Events	IR	Crude		Multivariable	
					HR	*P*	aHR*	*P*
					(95%CI)		(95%CI)	
All-cause Death	385	12.6	1,310	8.93	1.39	< 0.001	1.36	< 0.001
					(1.24–1.56)		(1.21–1.53)	
All-cause hospitalization	680	52.2	3,145	43.5	1.01	0.824	1.07	0.219
					(0.91–1.12)		(0.96–1.18)	
Infection-related admission	269	11.7	1,272	11	1.07	0.435	0.97	0.781
					(0.89–1.30)		(0.80–1.18)	
MACE	200	7.6	942	7.4	0.93	0.49	0.91	0.372
					(0.75–1.14)		(0.73–1.12)	

*Further adjusted comorbidities (hypertension, diabetes mellitus, coronary artery disease, congestive heart failure, atrial fibrillation, peripheral vascular disease, stroke, chronic obstructive pulmonary disease, hyperlipidemia, polycystic kidney) and medications (renin-angiotensin system blockades, beta-blocker, calcium channel blockers, anti-coagulants, dipeptidyl peptidase-4, and Lipid-lowering agents).

*PD, peritoneal dialysis; HD, hemodialysis; IR, incidence rate (every 100 person-years); HR, hazard ratio; aHR, adjusted hazard ratio; CI, confidence interval; MACE, major adverse cardiovascular events.*

**TABLE 3:** The causes of death in hemodialysis patients.

Groups	The cause of death							
	Cancer	Sepsis	CVD	DM	Liver	ESRD	Others	*P value*
Transitioned from PD [*n* (%)]	22 (5.7)	43 (11.2)	97 (25.2)	74 (19.2)	17 (4.4)	85 (22.1)	47 (12.2)	
								0.748
HD-only [*n* (%)]	76 (5.8)	152 (11.6)	315 (24)	264 (20.2)	51 (3.9)	326 (24.9)	126 (9.6)	

*HD, hemodialysis; PD, peritoneal dialysis; CVD, cardiovascular disease; DM, diabetes mellitus; ESRD, end stage of renal disease.*

As presented in Table 2, the patients who had transitioned from PD had a higher risk of all-cause death (HR: 1.39; 95% CI: 1.24–1.56) in crude analysis, and this significant association remained even after adjustment for comorbidities and drug use (HR: 1.36; 95% CI: 1.21–1.53). The parameter estimates for the covariates in the multivariable model of all-cause death are shown in Supplementary Table 3. Because of the assumption of proportional hazard was violated (time interaction, *p* < 0.001), the times axis were divided into four periods (≤5 months, 5–24 months, 2–5 years, and >5 years), a significant risk difference was observed only in the ≤5 months period (HR: 2.61; 95% CI: 2.04–3.35) (Table 4).

**TABLE 4:** Outcome analysis of mortality and hospitalization by times axis in hemodialysis patients.

	Transitioned from PD vs. HD-only					
			All-cause death		All-cause hospitalization	
Partition of time axis	Events of Death (%)^#^	Events of Hospitalization (%)^#^	aHR* (95%CI)	*P*	aHR* (95%CI)	*P*
≤5 _months	177/150	266/964	2.61	< 0.001	0.91	0.184
	(46 vs. 11)	(39 vs. 31)	(2.04–3.35)		(0.80–1.05)	
5–24 _months	93/495	299/1446	0.87	0.218	1.05	0.585
	(24 vs. 38)	(44 vs. 46)	(0.69–1.09)		(0.91–1.17)	
2–5 _years	88/507	102/634	0.85	0.164	0.91	0.37
	(23 vs. 39)	(15 vs. 20)	(0.67–1.07)		(0.73–1.12)	
> 5 _years	27/158	13/101	0.73	0.145	0.71	0.269
	(7 vs. 12)	(2 vs. 3)	(0.48–1.11)		(0.39–1.30)	

*Multivariable adjusting model was the same as that in Table 2.

*^#^The portion of deaths in the different time axis by total deaths.*

*HD, hemodialysis; PD, peritoneal dialysis; HR, hazard ratio; aHR, adjusted hazard ratio; CI, confidence interval.*

### MACE and Hospitalization Between the Groups

As presented in Table 2, the incidence of all-cause hospitalization, infection-related admission, and MACE between the transition from PD to HD and HD-only groups were 0.522/PY versus 0.435/PY, 0.117/PY versus 0.11/PY, and 0.076/PY versus 0.074/PY, respectively. The Kaplan–Meier event-free curves for all-cause hospitalization (χ^2^ = 33.02, *p* < 0.001), infection-related admission (χ^2^ = 0.22, *p* = 0.635) and MACE (χ^2^ = 0.07, *p* = 0.79) are displayed in Figures 2B–D.

As indicated by the crude analysis presented in Table 2, no significant differences in risk of all-cause hospitalization (HR: 1.01; 95% CI: 0.91–1.12), infection-related admission (HR: 1.07; 95% CI: 0.89–1.30), and MACE (HR: 0.93; 95% CI: 0.75–1.14) were identified between the groups. These associations remained non-significant even after further adjustment for comorbidities and drug use, with HRs of 1.07 (95% CI: 0.96–1.18), 0.97 (95% CI: 0.80–1.18), and 0.91 (95% CI: 0.73–1.12) for all-cause hospitalization, infection-related admission, and MACE, respectively.

A sensitivity analysis was done by removing the exclusion criteria of CABG. There was still no significant difference in risk of MACE between the transition from PD to HD and HD-only groups (HR: 0.95; 95% CI: 0.81–1.12). The assumption of proportional hazard for all-cause hospitalization was violated (*p* = 0.044). Therefore, the times axis were partitioned into four groups (≤5 months, 5–24 months, 2–5 years, and >5 years) and no significant differences in risk of all-cause hospitalization was noted in the different time periods (Table 4).

### Subgroup Analysis

**FIGURE 3:**
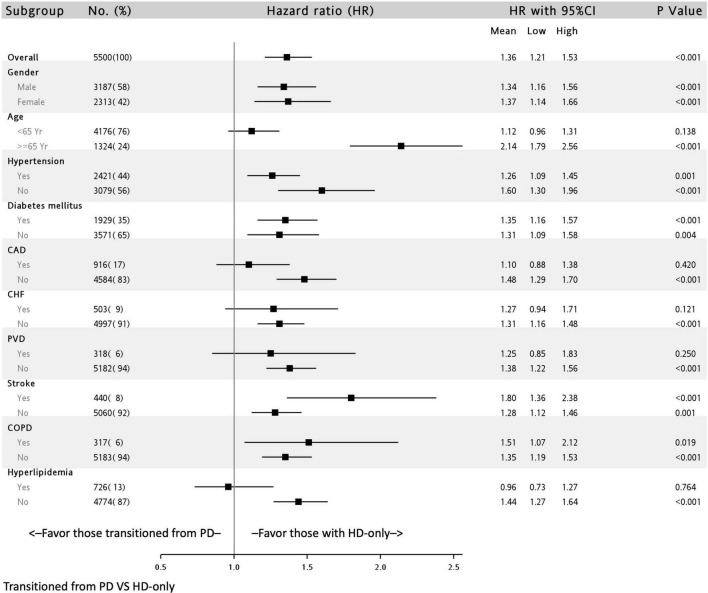
Subgroup analysis of all-cause mortality of patients undergoing dialysis with and without previous peritoneal dialysis in a multivariable adjusting model^∗^. ^∗^Full adjusted model is the same as that in Table 2. CAD, coronary artery disease; CHF, congestive heart failure; PVD, peripheral vascular disease; COPD, chronic obstructive pulmonary disease.

The association of all-cause deaths in the transition from PD to HD group stratified by covariates is presented in Figure 3. The hazardous outcomes in this group were consistent and significant in all subgroups, with the exception of the subgroup with patients aged < 65 years (HR: 1.12; 95% CI: 0.96–1.31) and with a history of CAD (HR: 1.10; 95% CI: 0.88–1.38), CHF (HR: 1.27; 95% CI: 0.94–.1.71), PVD (HR: 1.25; 95% CI: 0.85–1.83), and hyperlipidemia (HR: 0.96; 95% CI: 0.73–1.27).

In the patients with a DM history, the association of all-cause deaths in the transition from PD kept significant (HR: 1.35; 95% CI: 1.16–1.57), but the association of all-cause hospitalization, infection-related admission, and MACE didn’t (Supplementary Table 4).

## Discussion

Through analysis of data from a nationwide database, we demonstrated the outcomes of patients with ESRD who transitioned from PD to HD due to PD failure. The results indicate no significant differences in hospitalization and MACE in the transition from PD to HD group compared with the HD-only group. However, a higher rate of all-cause mortality was observed in the transition from PD to HD group, with the significance of this association remaining consistent in most subgroups. Moreover, the risk of mortality was noted to be the highest in the 5 months following dialysis transition. The results of our study not only expand on current understanding of dialysis but also indicate that greater care should be given to patients undergoing HD who have transitioned from PD.

Whether PD or HD is a more favorable treatment option for patients with ESRD has long been a topic of debate (16). Generally, PD offers more favorable preservation of residual renal function (17), quality of life (18), and stability of hemodynamic changes than HD (19). In addition, the survival outcomes did not seem to differ between PD and HD (20, 21). Accordingly, PD has been identified as a favorable first treatment choice. However, such favorability was not indicated in a small randomized controlled trial conducted by Korevaar JC et al. (22). Therefore, clinicians should view both PD and HD as viable options for treating ESRD.

Transitioning from PD to HD is not uncommon during the disease course among patients with dialysis-dependent ESRD. The average mortality-censored medial technique survival for this group is only 3.7 years (11). In a report that used data from the NECOSAD Study Group, the 2-year PD technique survival was 64%, indicating that one-third of the patients had dropped out of the treatment (23). Transition from PD to HD is a common and gradual process in patients with ESRD who undergo PD as their first dialysis modality (11). Jaar et al. (24) conducted a prospective study of 262 patients and reported no difference in survival between a PD group that transitioned to HD and a PD group that did not (HR, 0.89; 95% CI: 0.41–1.93). Chen et al. by using Australian and New Zealand Dialysis and Transplant (ANZDATA) registry data, reported infection and social-related technique failure to be associated with deaths within 2 years of technique failure in patients undergoing PD (25). To our knowledge, no study has compared the outcomes of transitioning from PD to HD with those of HD-only.

In our study, transitioning from PD to HD yielded the same clinical outcomes as (all-cause hospitalization, infection-hospitalization, and MACE) but poorer survival outcomes than HD-only. Furthermore, the piecewise HR showed that such worse outcome occurred especially in the first 5 months after transitioning to HD. This is possibly because the early period after transitioning to HD are key with respect to premature mortality in such a group, which is consistent with the results of Chen et al. (25). Therefore, a personalized care program for patients undergoing HD who have transitioned from PD may be warranted to prevent mortality.

In the subgroup analysis, we found that older adults had the poorest mortality outcomes (HR: 2.14; 95% CI, 1.79–2.56). Dialysis in older patients presents several challenges; the choice of dialysis modality for older patients is mostly dependent on caregiver (such as family) preferences. In the literature, whether mortality is higher in PD than in HD is highly debated. Several reports have indicated no difference in mortality between PD and HD in older patients (26, 27). However, some studies have reported that the mortality rate of patients undergoing PD was higher than that of those undergoing HD (28, 29). In a study conducted by Sakai K et al. older adults undergoing PD had similar rates of peritonitis or catheter-related complications but lower rates of transition to HD compared with adult patients undergoing PD (30). This may explain our findings. The conditions and complications of transitioning to HD may have been severe for the older adults included in our study, which may have led to poorer outcomes in the older adults in this group than in those in the HD control group. Moreover, in our study, the association was non-significant in patients with severe comorbidities, such as CAD, CHF, PVD, and hyperlipidemia. These comorbidities may, therefore, be hypothesized to play a major role in mortality in older adult populations transitioning from PD to HD. Atherosclerosis and stenosis of the large vessels could result in reduced blood flow to the smaller arteries and arterioles of the peritoneum leading to reduced ultrafiltration and clearance, which resultantly to PD technique failure (31). In addition to the above hypothesis, the other possible explanation for these insignificant results is the small sample size of these subgroups in our study.

Notably, we also found that patients with prior stroke had higher mortality rates when they had transitioned from PD to HD (HR: 1.80; 95% CI: 1.36–2.38). According to a retrospective study by Wu et al. patients who underwent continual ambulatory PD with prior stroke had a higher rate of all-cause mortality and death-censored technique failure compared with those without prior stroke. The study further reported older age and nutritional status to be independent risk factors (32). In this cohort study, patients with prior stroke undergoing PD had higher rates of peritonitis, leading to a need for transition to HD. Malnutrition has been well documented as a risk factor for mortality in PD (33, 34). Patients who have experienced previous stroke are prone to malnutrition (35). This may be due to underlying chronic inflammation, infection, and an inability to achieve optimal intake. In our cohort, patients with stroke transitioning from PD to HD had higher mortality rates, suggesting that these two factors have additive effects, which may be caused by malnutrition.

Our study had several strengths. First, this was a nationwide population-based cohort study (real-world evidence), which ensured a large sample size and the generalizable results. Second, our follow-up duration was sufficiently long to obtain primary outcomes. Finally, we used propensity scores to remove baseline imbalances and adjusted for potential confounders, which improved the reliability of our inferences. However, our study also has some limitations. First, data on potential confounding factors, such as body mass index, blood pressure, laboratory data, lifestyle, and dialysis quality, were not available in the NHI database. However, the consistency in the majority of the subgroup analyses indicates that our study results are robust. Second, the reasons for PD failure were also unavailable in the NHI database, which prevented us from conducting further risk analyses for deaths in patients who had undergone PD. Chen et al. reported that infection and social-related PD technique failure are associated with premature death within 2 years in patients who transition from PD to HD (25). Therefore, further study of risk assessment in such a population is required. Third, the medication use between groups was not balanced. The more prescription of RASB, beta-blockers, CCB and anticoagulants in the group transitioned from PD to HD might indicate volume overload, which lead to higher mortality. Nevertheless, further multiple variables adjusted in the data analysis can reduce this bias. Finally, the results derived from this Taiwanese population of patients undergoing HD may require further verification to be applied to other ethnic populations.

## Conclusion

Patients transitioning from PD to HD may experience poorer mortality outcomes compared with patients who are HD-only, especially within 5 months of the transition. Although this finding does not evidence the superiority of either PD or HD, it indicates that clinicians transitioning patients from PD to HD should be well trained and well prepared.

## Data Availability Statement

The dataset used in this study is held by the Taiwan Ministry of Health and Welfare (MOHW). Due to the General Data Protection Regulation, the dataset is not available on request from the corresponding author. Any researcher interested in accessing this dataset can apply for access. The MOHW will evaluate the application for accessing this dataset. Please visit the website of the National Health Informatics Project of the MOHW (https://dep.mohw.gov.tw/dos/np-2497-113.html).

## Ethics Statement

The studies involving human participants were reviewed and approved by Institutional review board of Shin-Kong Wu Ho-Su Memorial Hospital. Written informed consent for participation was not required for this study in accordance with the national legislation and the institutional requirements.

## Author Contributions

M-HT, T-NJ, and Y-WF: conceived and designed the experiments. Y-YC and J-TW: performed the experiments. Y-YC: analyzed the data. M-HT and Y-WF: wrote the manuscript. All authors contributed reagents, materials, and analysis tools and approved the manuscript.

## Conflict of Interest

The authors declare that the research was conducted in the absence of any commercial or financial relationships that could be construed as a potential conflict of interest.

## Publisher’s Note

All claims expressed in this article are solely those of the authors and do not necessarily represent those of their affiliated organizations, or those of the publisher, the editors and the reviewers. Any product that may be evaluated in this article, or claim that may be made by its manufacturer, is not guaranteed or endorsed by the publisher.
