# Supplementary material for: Outcome Analysis of Transition From Peritoneal Dialysis to Hemodialysis: A Population-Based Study
Source: Front Med (Lausanne). 2022 Jun 2;9:876229. doi: 10.3389/fmed.2022.876229 (PMC9202657; doi:10.3389/fmed.2022.876229)
Supplement: Supplementary file 1 [file Table_1.DOCX]

| **S1. Code for diseases** | | |
| --- | --- | --- |
|  | ICD-9-CM | ICD-10-CM |
| ***Comorbidities*** | | |
| Hypertension | 401 – 405 | I10–14 |
| Diabetes mellitus | 250 | E10–E14 |
| Coronary artery disease | 410 – 414 | I20–I25 |
| Congestive heart failure | 428.0, 428.1, 428.9 | I50 |
| Atrial fibrillation | 427.31 | I48.0, I48.2, I48.91 |
| Peripheral vascular disease | 250.7, 440.2-3,440.8-9,  443,444.22,444.8,447.8-9 | E08.51-52, E08.59, E09.51-52, E09.59, E10.51-52, E10.59, E11.51-52, E11.59, E13.51-52, E13.59, I70.2-I70.9, I73, I74.2-I74.9, I75.011-I75.029, I75.89, I77.3, I77.89, I77.9, I79.1, I79.8 |
| Stroke | 430–438 | I60- I63, I65-I69, G45-G46 |
| COPD | 490–492, 496 | J40–J44, J47 |
| Hyperlipidemia | 272.0–272.4 | E78 |
| Polycystic kidney | 753.12–753.14 | Q61.1, Q61.19, Q61.2, Q61.3 |
| ***CCI score*** | | |
| Myocardial infarct | 410 | I21–22 |
| Congestive heart failure | 428.0, 428.1, 428.9 | I50 |
| Cerebrovascular disease | 430–438 | I60–I63, I65–I69, G45-G46 |
| Connective tissue disease | 710,714,725 | M05, M06, M32, M33.20, M33.29, M34, M35.3 |
| Peripheral vascular disease | 250.7, 440.2-3,440.8-9,  443,444.22,444.8,447.8-9 | E08.51-52, E08.59, E09.51-52, E09.59, E10.51-52, E10.59, E11.51-52, E11.59, E13.51-52, E13.59, I70.2-I70.9, I73, I74.2-I74.9, I75.011-I75.029, I75.89, I77.3, I77.89, I77.9, I79.1, I79.8 |
| Dementia | 290 | F03.90, F05, F01.50,F01.51 |
| COPD | 490–492, 496 | J40–J44, J47 |
| Ulcer disease | 531.XX, 532.XX, 533.XX ,534.XX | K25.X,K26.X,K27.X,K28.X,K31.82,K56.60 |
| Mild liver disease | 571.2,571.4X,571.5,571.6 | K70.2,K70.3X, K73.X,K74.X,K74.60,K74.69,K75.4 |
| Hemiplegia | 342 | G81 |
| Moderate-to-severe renal disease | 582.0,582.1,582.2,582.4,582.81,582.89,582.9,583.0,  583.1,583.2,583.4,583.6,583.7,583.9,588.1,588.8,  588.9, 583.81,583.89,585,586,588 | E10.21, E11.21, N03.0, N03.1, N03.2, N03.3, N03.4,  N03.5 N03.6, N03.7, N03.8, N03.9, N05.0, N05.1,  N05.2, N05.3, N05.4, N05.5, N05.6, N05.7, N05.8, N05.9, N06.0, N06.1, N06.2, N06.3, N06.4, N06.5, N06.6, N06.7, N06.8, N06.9, N07.0, N07.1, N07.2, N07.3, N07.4, N07.5, N07.6, N07.7, N07.8, N07.9,  N08, N14.0, N14.1, N14.2, N14.3, N14.4, N15.0, N15.8,  N15.9, N16, N17.1, N17.2, N18.4, N18.5, N18.6, N18.9,  N19, N25.0, N25.1, N25.81, N25.89, N25.9 |
| Diabetes-with-end-organ-damage | 250.4,250.5,250.6 | E08.311, E08.319, E08.321, E08.329, E08.331, E08.339,  E08.341, E08.349, E08.351, E08.359, E08.36, E08.39,  E09.311, E09.319, E09.321, E09.329, E09.331, E09.339,  E09.341, E09.349, E09.351, E09.359, E09.36, E09.39,  E10.21, E10.22, E10.29, E10.311, E10.319, E10.36,  E10.39, E10.40, E10.41, E10.44, E10.49, E10.610,  E10.65, E11.21, E11.22, E11.29, E11.311, E11.319,  E11.321, E11.329, E11.331, E11.339, E11.341, E11.349,  E11.351, E11.359, E11.36, E11.39, E11.40, E11.41,  E11.42, E11.43, E11.44, E11.49, E11.65, E13.21, E13.22, E13.29, E13.311, E13.319, E13.321, E13.329,  E13.331, E13.339, E13.341, E13.349, E13.351, E13.359,  E13.36, E13.39, E13.42, E13.43, E13.44, E13.49 |
| Malignancy | 140-149,150-159,160-165,170,174,175,176,179,180-189,190-195,200-201,203-208 | C00–C96,D45, D47.Z9, E31.22, Z51.12 |
| Moderate or severe liver disease | 456.0,456.1,456.20,572.X,456.21,I85.XX, | K72.10,K72.11,K72.90,K72.91,K766,K767,K768.1 |
| Metastatic solid tumors | 196.X,197.X,198.X, 198.8X,199.X | C45.9, C77.X, C78, C79, C7A.00, C7A.094, C7A.095,  C7A.096, C7A.1, C7A.8, C7B, C80.0, C80.1, D3A.8,  J91.0, R18.0, Z51.12 |
| AIDS | 0.42,0.43,0.44 | B20 |
| ***Outcomes*** | | |
| Infection-related admission | 008.45, 038.XX, 040.0, 070, 421.X, 480.X, 481.X, 482.X, 483.X, 484.X, 485, 486, 487.0, 487.1, 513, 562.11, 566, 567.0, 567.1, 567.2, 567.9, 569.5, 574.0X, 574.1X, 574.3X, 574.4X, 574.6X, 574.7X, 574.8X, 575.0, 575.1X, 590.1X, 590.2, 590.80, 590.9, 599.0, 681.00, 681.10, 681.9, 682.X, 711.0X, 711.9X, 728.86, 730.0X, 730.2X, 785.4, 790.7 | A04.7, A22.1, A37.91, A40.0-A41.9, A48.0, A48.1, B15.X- B19.X, B25.0, B44.0, I33.X, I39, I96, J09.X1, J09.X2, J10.0X, J10.1, J11.0X, J11.1, J12.X- J17, J18.0, J18.1, J18.8, J18.9, J85.X, K12.2, K57.20, K57.32, K57.40, K57.52, K57.92, K61.X, K63.0, K65.0-K65.2, K65.8, K65.9, K67, K68.11, K68.19, K68.9, K80.XX, K81.X, L02, L03, L98.3, M00, M01.X0, M01.X19, M01.X29, M01.X39, M01.X49, M01.X59, M01.X69, M01.X79, M01.X8, M01.X9, M46.2X, M72.6, M86.0X, M86.1X, M86.2X, M86.9, N10, N11.9, N12, N13.6, N15.1, N15.9, N39.0, R65.XX, R78.81 |
| MACE | 410,426-427,428,430-438,437 | I121-I122, I44-I49, I50, I60-I63, I65-I69 |
| ***The causes of death*** | | |
| Cancer | 140–208 | C00–C97 |
| Sepsis | 001–009,480–486 | A00–A09, J12–J18,J40–J47 |
| CVD | 401–405,390–392,393–398,410–414,420–429,430–438,440 | I10–I15,I01–I02.0,I05–I09,I20–I25,I27,I30–I52,I60–I69,I70,I71 |
| DM | 250 | E10-E14 |
| Liver | 571 | K70,K73–K74,K80–K82 |
| ESRD | 580–589 | N00–N07,N17–N19,N25–N27 |
| Abbreviation:  ICD, international classification of disease; COPD, chronic obstructive pulmonary disease; CCI, charlson comorbidity index; AIDS, acquired immune deficiency syndrome; MACE, major adverse cardiac events; CVD, cardiovascular disease; DM, diabetes mellitus; ESRD, end stage of renal disease. | | |
