# Supplementary material for: Outcome Analysis of Transition From Peritoneal Dialysis to Hemodialysis: A Population-Based Study
Source: Front Med (Lausanne). 2022 Jun 2;9:876229. doi: 10.3389/fmed.2022.876229 (PMC9202657; doi:10.3389/fmed.2022.876229)
Supplement: Supplementary file 2 [file Table_2.DOCX]

| **S2. Code for drugs** | | |
| --- | --- | --- |
| Drug type | ATC classification system codes | Drug name |
| RASB | C09A, C09B, C09C, C09D | Captopril, Enalapril, Lisinopril, Perindopril, Ramipril, Quinapril, Benazepril, Cilazapril, Fosinopril, Imidapril, Losartan, Eprosartan, Valsartan, Irbesartan, Candesartan, Telmisartan, Olmesartan, Azilsartan |
| Beta blocking agents | C07A, C07B, C07CA03, C07DA06 | Alprenolol, Oxprenolol, Pindolol, Propranolol, Timolol, Sotalol, Nadolol, Carteolol, Bupranolol, Metoprolol, Atenolol, Acebutolol, Betaxolol, Bevantolol, Bisoprolol, Esmolol, Nebivolol, Labetalol, Carvedilol |
| CCB | C08C, C08D, C08E, C09BB, C09DB,  C09DX, C10BX | Amlopidine, Felodipine, Isradipine, Nicardipine, Nifedipine, Nimodipine, Nisoldipine, Nitrendipine, Lacidipine, Barnidipine, Lercanidipine, Cilnidipine, Benidipine, Verapamil, Diltiazem |
| Anti-coagulants | B01AA, B01AB, B01AC, B01AD, B01AE, B01AF, B01AX | Phenindione, warfarin, heparin, dalteparin, enoxaparin, nadroparin, tinzaparin, clopidogrel, ticlopidine, acetylsalicylic acid, dipyridamole, epoprostenol, iloprost, Abciximab, eptifibatide, tirofiban, Treprostinil, prasugrel, cilostazol, ticagrelor,  Selexipag, combinations, Cilostazol, streptokinase, alteplase, urokinase, drotrecogin alfa (activated), Tenecteplase, dabigatran etexilate, rivaroxaban,apixaban, edoxaban, fondaparinux |
| DPP4 | A10BH | Sitagliptin, Vildagliptin, Saxagliptin, Alogliptin, Linagliptin |
| Lipid-lowering agents | C10AA, C10BA, C10BX03 | Simvastatin, Lovastatin, Pravastatin, Fluvastatin, Atorvastatin, Rosuvastatin, Pitavastatin |
| Abbreviation:  RASB, renin-angiotensin system blockades; CCB, calcium channel blockers; DPP4, dipeptidyl peptidase-4. | | |
