# Supplementary material for: Outcome Analysis of Transition From Peritoneal Dialysis to Hemodialysis: A Population-Based Study
Source: Front Med (Lausanne). 2022 Jun 2;9:876229. doi: 10.3389/fmed.2022.876229 (PMC9202657; doi:10.3389/fmed.2022.876229)
Supplement: Supplementary file 3 [file Table_3.docx]

| S3. The parameter estimates for the covariates in the multivariable model | | | | |
| --- | --- | --- | --- | --- |
| Parameters | All-cause death | | All-cause hospitalization | |
|  | HR (95%CI) | *P* |  | *P* |
| Transitioned from PD  (vs HD-only) | 1.36 (1.21–1.53) | <0.001 | 1.07 (0.96–1.18) | 0.219 |
| **Comorbidities** |  |  |  |  |
| Hypertension | 0.94 (0.83–1.07) | 0.354 | 1.01 (0.91–1.12) | 0.219 |
| Diabetes mellitus | 1.80 (1.60–2.01) | <0.001 | 0.81 (0.73–0.89) | 0.874 |
| CAD | 1.31 (1.16–1.48) | <0.001 | 0.90 (0.80–1.02) | <0.001 |
| CHF | 1.32 (1.15–1.52) | <0.001 | 0.80 (0.68–0.94) | 0.107 |
| Atrial fibrillation | 1.79 (1.35–2.36) | <0.001 | 0.84 (0.58–1.22) | 0.005 |
| PVD | 1.10 (0.94–1.30) | 0.243 | 0.98 (0.82–1.18) | 0.357 |
| Stroke | 1.87 (1.63–2.14) | <0.001 | 0.64 (0.53–0.77) | 0.832 |
| COPD | 1.48 (1.26–1.73) | <0.001 | 0.78 (0.64–0.94) | <0.001 |
| Hyperlipidemia | 0.86 (0.75–1.00) | 0.049 | 0.96 (0.84–1.10) | 0.008 |
| Polycystic kidney | 0.80 (0.30–2.14) | 0.652 | 1.33 (0.71–2.49) | 0.570 |
| **Prescriptions** |  |  |  |  |
| RASB | 1.01 (0.89–1.13) | 0.919 | 0.97 (0.87–1.08) | 0.374 |
| Bata-blocker | 0.83 (0.74–0.93) | 0.001 | 1.08 (0.97–1.20) | 0.538 |
| CCB | 1.00 (0.88–1.13) | 0.991 | 0.88 (0.79–0.98) | 0.162 |
| Anticoagulants | 1.33 (1.20–1.49) | <0.001 | 0.90 (0.81–100) | 0.023 |
| DPP4 | 1.12 (0.97–1.31) | 0.127 | 0.96 (0.82–1.13) | 0.047 |
| Lipid-lowering agents | 0.86 ( 0.76–0.98) | 0.025 | 1.17 (1.04–1.31) | 0.631 |
| Abbreviation: HD, Hemodialysis; PD, Peritoneal Dialysis; CAD, coronary artery disease; CHF, congestive heart failure; PVD, peripheral vascular disease; COPD, chronic obstructive pulmonary disease; RASB, Renin-Angiotensin System blockades; CCB, calcium channel blockers; DPP4, dipeptidyl peptidase-4 | | | | |
