# Supplementary material for: Outcome Analysis of Transition From Peritoneal Dialysis to Hemodialysis: A Population-Based Study
Source: Front Med (Lausanne). 2022 Jun 2;9:876229. doi: 10.3389/fmed.2022.876229 (PMC9202657; doi:10.3389/fmed.2022.876229)
Supplement: Supplementary file 4 [file Table_4.docx]

| **S4. Outcomes between hemodialysis patients with/without previous peritoneal dialysis in the diabetic subgroup** | | |
| --- | --- | --- |
| Outcomes | Transitioned from PD vs. HD-only | |
|  | aHR* (95%CI) | *P value* |
| All-cause Death | 1.35 (1.16­–1.57) | <0.001 |
| All-cause hospitalization | 0.99 (0.87–1.12) | 0.834 |
| Infection-related admission | 0.94 (0.79–1.11) | 0.439 |
| MACE | 0.97 (0.82–1.1) | 0.693 |
| *Multivariable adjusting model was the same as that in table 2.  Abbreviation: HD, hemodialysis; PD, peritoneal dialysis; aHR, adjusted hazard ratio; CI, confidence interval; MACE, major adverse cardiovascular events. | | |
